# Design and Simulation of Efficient SnS-Based Solar Cell Using Spiro-OMeTAD as Hole Transport Layer

**DOI:** 10.3390/nano12142506

**Published:** 2022-07-21

**Authors:** Pooja Tiwari, Maged F. Alotaibi, Yas Al-Hadeethi, Vaibhava Srivastava, Bassim Arkook, Sadanand, Pooja Lohia, Dilip Kumar Dwivedi, Ahmad Umar, Hassan Algadi, Sotirios Baskoutas

**Affiliations:** 1 Department of Electronics and Communication Engineering, Madan Mohan Malviya University of Technology, Gorakhpur 273010, India; poojatiwari23@gmail.com (P.T.); me.vaibhava@gmail.com (V.S.); 2 Department of Physics, Faculty of Science, King Abdulaziz University, Jeddah 21589, Saudi Arabia; malhabrdi@kau.edu.sa (M.F.A.); yalhadeethi@kau.edu.sa (Y.A.-H.); barkook@kau.edu.saa (B.A.); 3 Department of Physics and Astronomy, University of California, Riverside, CA 92507, USA; 4 Photonics and Photovoltaic Research Lab, Department of Physics and Material Science, Madan Mohan Malviya University of Technology, Gorakhpur 273010, India; 5 Department of Applied Science, World College of Technology and Management, Gurugram 122506, India; 6 Department of Chemistry, College of Science and Arts, Promising Centre for Sensors and Electronic Devices (PCSED), Najran University, Najran 11001, Saudi Arabia; 7 Department of Materials Science and Engineering, The Ohio State University, Columbus, OH 43210, USA; 8 Department of Electrical Engineering, College of Engineering, Najran University, Najran 11001, Saudi Arabia; hassan.algadi@gmail.com; 9 Department of Materials Science, University of Patras, 26504 Patras, Greece; bask@upatras.gr

**Keywords:** Spiro-OMeTAD, SnS, CeO_2_, heterojunction solar cell, HTL (hole transport layer)

## Abstract

In the present paper, the theoretical investigation of the device structure ITO/CeO_2_/SnS/Spiro-OMeTAD/Mo of SnS-based solar cell has been performed. The aim of this work is to examine how the Spiro-OMeTAD HTL affects the performance of SnS-based heterostructure solar cell. Using SCAPS-1D simulation software, various parameters of SnS-based solar cell such as work function, series and shunt resistance and working temperature have been investigated. With the help of Spiro-OMeTAD, the suggested cell’s open-circuit voltage was increased to 344 mV. The use of Spiro-OMeTAD HTL in the SnS-based solar cell resulted in 14% efficiency increase, and the proposed heterojunction solar cell has 25.65% efficiency. The cell’s performance is determined by the carrier density and width of the CeO_2_ ETL (electron transport layer), SnS absorber layer and Spiro-OMeTAD HTL (hole transport layer). These data reveal that the Spiro-OMeTAD solar cells could have been a good HTL (hole transport layer) in regards to producing SnS-based heterojunction solar cell with high efficiency and reduced cost.

## 1. Introduction

Thin-film solar cells are a popular field in the scientific world. The solar cell economy is rapidly changing and progressing; economically viable thin-film solar cells is a new problem. Production of material cost competitiveness in thin-film solar cells is no longer possible when compared to wafer-based technologies, and new technologies are required to enhance efficiency and reduce cost. Taking this into account, SnS (orthorhombic tin monosulphide) has become an important absorbent layer of thin-film solar cells [1,2,3,4,5,6,7,8]. SnS offer several benefits, including an energy band gap of 1.31 eV and a larger absorption coefficient of 10^5^ cm^−1^, allowing SnS layers to absorb a large portion of the solar spectrum [9]. Furthermore, SnS-based solar cell is a non-toxic and abundant material that may be produced at a minimal price using dip coating, spin coating and spray pyrolysis methods [10,11,12,13,14]. A number of functional SnS-based heterojunction solar cells have been described in recent years [15,16,17]. At present, the ZnS/SnS heterostructure has maximum efficiency of 16.26% [18]. To fabricate a device, having high efficiency at low cost, CeO_2_ as an ETL is an appropriate choice for SnS-based solar cells because of the large tunable bandgap (3.0–3.6 eV) and superior electrical and optical properties [19,20]. A simple, low-cost sol-gel dip coating and spin coating process can be used to synthesize CeO_2_ [21]. According to Tan et al., nanostructured CeO_2_/Al back electrode could improve the light trapping of the solar cells [19].

Further, in this study Spiro-OMeTAD is used as HTL (hole transport layer) material due to its ease of fabrication and high performance [22,23,24]. It possesses a high glass transition temperature (Tg), so it is simple to deal with, having stable morphology while maintaining all electrical properties. The post-annealing treatment is not required for Spiro-OMeTAD and it has large solubility as well. For SnS-based solar cell, Spiro-OMeTAD is a good HTL because of its good band alignment with SnS.

Numerical simulation of a photovoltaic device would be required to determine the optimum parameters of a model and assess the effect of physical parameters on model performance. The simulation method is validated by comparing simulation results to experimental data. Herein, the device is simulated by SCAPS-1D program, and the influence of various structural properties of SnS layer, CeO_2_ layer and Spiro-OMeTAD layer on the performance of SnS-based solar cell has been thoroughly examined. The modelling and advancement of a unique device structure (ITO/CeO_2_/SnS/Spiro-OMeTAD/Mo) of an SnS-based solar cell using the CeO_2_ electron transport layer and Spiro-OMeTAD HTL have been proposed.

The goal of this research is to demonstrate that the efficiency of a solar cell can be enhanced by trapping incident light on its surface and optimizing the various parameters of the device structure.

## 2. Structure and Material Properties of Solar Cell

**Figure 1:**
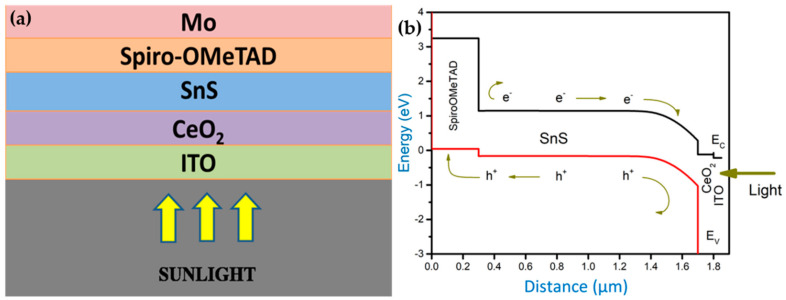
(**a**) Schematic layout of the proposed heterostructure solar cell and (**b**) the energy band diagram of the proposed heterostructure solar cell.

The suggested ITO/CeO_2_/SnS/Spiro-OMeTAD/Mo solar cell schematic structure is shown in Figure 1a. ITO serves as a front contact TCO, CeO_2_ works as the window layer, Spiro-OMeTAD operates as the HTL, SnS acts as the absorber layer and Mo serves as the rear contact. The suggested structure’s energy band diagram (Figure 1b) reveals that the conduction band of the absorber layer SnS is less than that of the CeO_2_ ETL layer_,_ and the conduction band offset (CBO) between SnS and CeO_2_ layer is very small. As a result, electrons can flow from SnS to ITO easily through CeO_2_. So, the electrons can flexibly move through CeO_2_ from SnS to ITO. In between the absorber layer SnS and the ETL layer CeO_2_, there is a valance band offset (VBO) which is extremely high. As a result, the holes at ETL layer CeO_2_ will be blocked. The valance band of Spiro-OMeTAD HTL is higher than that of absorber layer SnS as shown in Figure 1b, and the valence band offset between these two layers is substantially smaller. In addition, the CBO between HTL layer Spiro-OMeTAD and absorber layer SnS is quite large, preventing electrons from SnS from reaching the back electrode. Table 1 lists the physical parameters for the simulations of ITO/CeO_2_/SnS/Spiro-OMeTAD/Mo heterostructure solar cell [20], whereas Table 2 lists the physical parameters of defect density present in SnS.

## 3. Results and Discussion

### 3.1. CeO_2_/SnS Solar Cell Open-Circuit Voltage

**Figure 2:**
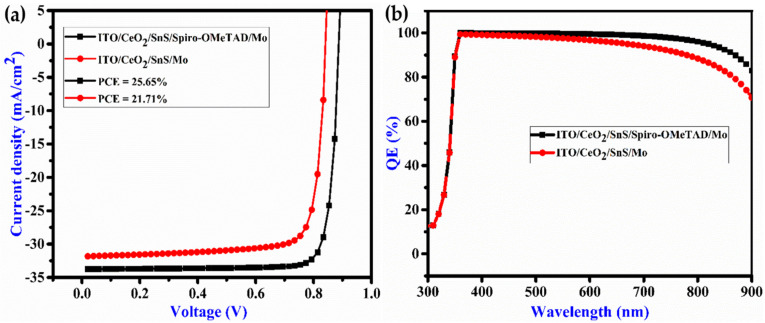
(**a**) J-V curve and (**b**) quantum efficiency of a proposed solar cell.

In solar cell, V_OC_ (open-circuit voltage) is determined by the dark current density. For thin-film solar cell, it can be computed as:

J_O_ = qn_i_^2^S/N_D_ (1)

where q denotes the charge, N_D_ denotes ETL doped concentration, S denotes radiative recombination rate at the absorber/electrode interface and n_i_ denotes absorber material’s inherent carrier density. Hence, the dark current at the absorber/electrode contact is proportional to the surface recombination rate. As an outcome, the metallization of the absorber surface is crucial for improving both V_OC_ and the performance of the cell. Figure 2a,b display the J-V curve and QE curve with and without Spiro-OMeTAD, respectively. The cell without Spiro-OMeTAD has V_OC_ of 0.8421 V, FF of 80.98%, J_sc_ of 31.83 mA/cm^2^ and PCE of 21.71% and with Spiro-OMeTAD V_OC_ is 0.8878 V, FF is 85.61%, J_sc_ is 33.74 mA/cm^2^ and PCE is 25.65%. As a result, the Spiro-OMeTAD HTL enhances the (V_OC_) voltage level as well as the PCE of the proposed solar cell. Due to excessive band offset, the Spiro-OMeTAD HTL appropriate band alignment will extend high potential, increasing the V_OC_. Figure 2b illustrates the Spiro-OMeTAD HTL, magnifying the quantum efficiency of the solar cell.

### 3.2. Effect of Spiro-OMeTAD/SnS Layers Interface on Defect Density

The role of the Spiro-OMeTAD/SnS interface on the performance of the solar cell was analyzed. The defect density varied from 10^10^ to 10^18^ cm^−2^, and the findings demonstrate that the density of interface defect of Spiro-OMeTAD/SnS has no impact on the performance of the solar cell till 10^16^ cm^−2^. With a further increase in the defect density above 10^16^ cm^−2^, the performance of the solar cell reduces marginally which can be solved by increasing the thickness of SnS layer.

### 3.3. Interface Defect Density of CeO_2_/SnS Layers

The probability of defect at CeO_2_/SnS interface varied from 10^10^ to 10^18^ cm^−2^. This defect increases the recombination rate due to which a decrease in V_OC_ is observed. Series resistance also increases due to which fill factor decreases. As a result, the high CeO_2_/SnS defect is to blame for higher series resistance. This reveals that the density of CeO_2_/SnS defects has a huge influence on solar cell performance. The structural imperfections of the two materials as well as metal cation passage through the absorbent layer during cell manufacturing cause interfacial defects [18].

### 3.4. Working Temperature and Back Contact Metal Work Function

**Figure 3:**
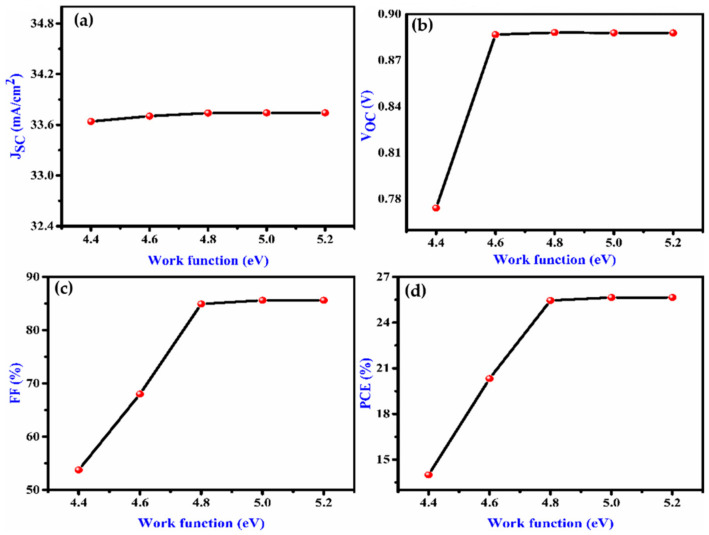
Effect on (**a**) J_SC_, (**b**), V_oc_ (**c**) FF (**d**) and PCE with variation of work function (eV) of the proposed device.

The effect of WF (work function) on the performance of the cell is evaluated as shown in Figure 3a–d. As the WF rises, V_OC_, FF and PCE also increase, up to a particular value of WF. This specifies that with higher WF, the majority of carrier barrier height decreases. As a result, WF has a profound impact on the performance of the solar cell. To achieve high efficiency, a suitable metal contact is required.

**Figure 4:**
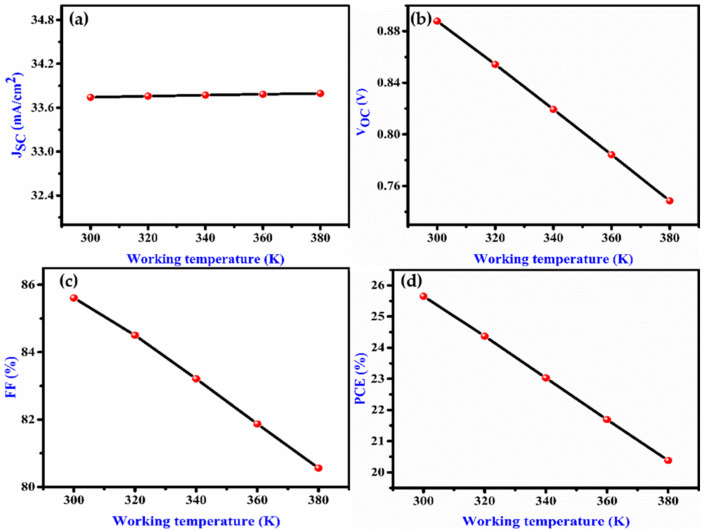
Effect on (**a**) J_SC_, (**b**)V_OC_, (**c**) FF and (**d**) PCE with variation of temperature of the proposed device.

If we increase the temperature of a semiconductor device, the velocity of the charge carrier increases and the band energy decreases. The reduction in band energy results in the reduction of the bandgap. At high temperatures, there will be a loss of power. As shown in Figure 4a–d, J_SC_ is unaffected by working temperature, whereas V_OC_ decreases as working temperature rises. Therefore, increasing working temperature lowers the solar cell efficiency.

### 3.5. Series Resistance and Shunt Resistance

**Figure 5:**
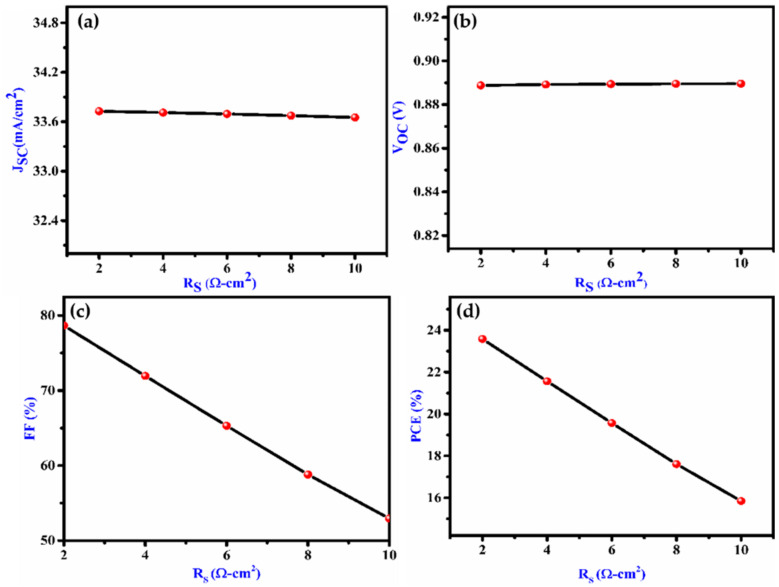
Effect on (**a**) J_SC_, (**b**) V_OC,_ (**c**) FF and (**d**) PCE with variation of series resistance of the proposed device.

The R_s_ and R_sh_ have a great influence on the performance of solar cells. It rises from the metal contacts of layers and solar cell. The functioning of the device was evaluated by altering the resistance from 2 to 25 cm^2^. Figure 5a–d illustrates that the V_OC_ and J_SC_ are unaffected by R_S_; however, the FF and PCE drop as R_s_ is increasing causes power dissipation.

**Figure 6:**
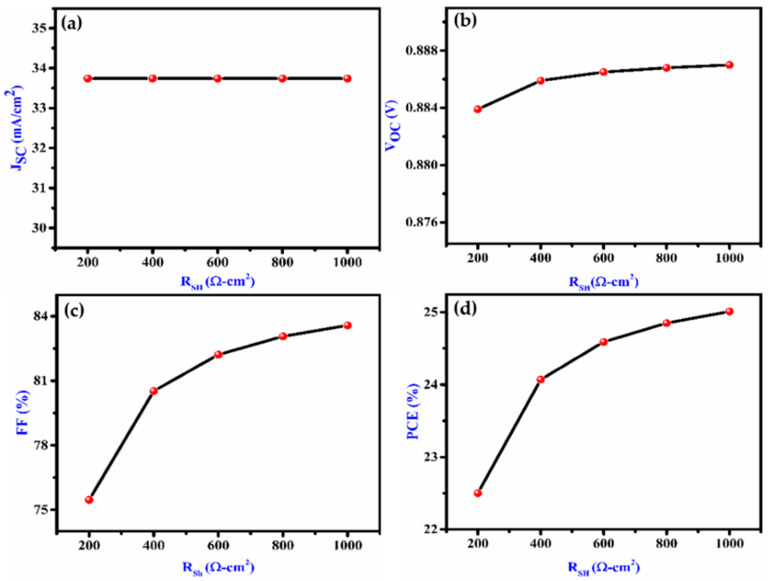
Effect on (**a**) J_SC_, (**b**) V_OC,_ (**c**) FF and (**d**) PCE with variation of shunt resistance of the proposed device.

Manufacturing flaws are the cause of R_sh_. The shunt resistance was altered from 100 to 1000 cm^2^. V_OC_ reduces when R_sh_ lowers because of the reduction of current to R_sh_. As seen in Figure 6a–d, R_sh_ > 600 Ω cm^2^ has very little effect on PCE. As a result, low shunt resistance has a huge impact on the performance of the cell.

### 3.6. Layer Thicknesses and Carrier Concentration of SnS, CeO_2_ and Spiro-OMeTAD Layer Optimization

The performance of the cell was evaluated in terms of layer thickness and carrier concentration. Fill factor and efficiency of 85.61% and 25.65% are established at Spiro-OMeTAD and CeO_2_ thicknesses as well as carrier concentration of 100 nm and 10^−21^ cm^−3^ accordingly.

The reduction of photogenerated charge carrier recombination is indicated by V_OC_ increasing slightly with Spiro-OMeTAD carrier concentration. At Spiro-OMeTAD thickness and carrier concentration of >100 nm and 10^16^ cm^−3^, FF and PCE were found to decrease with thickness but were unaffected by carrier concentration. Whereas, at Spiro-OMeTAD carrier concentration > 10^16^ cm^−3^, FF and PCE increase with carrier concentration but are unaffected by thickness. 

In cases of CeO_2_-when the thickness increases, V_OC_ drops but increases as the carrier concentration rises. By CeO_2_ thickness, FF is unaffected, although it rises in proportion to concentration of CeO_2_.

The primary influential criteria in designing solar cells are carrier concentration and absorber layer thickness. To achieve maximal device efficiency, both the functions must be maintained. To test the working of the cell, the concentration and thickness are tuned from 10^11^ to 10^21^ cm^−3^ and 200 to 2000 nm, respectively. At SnS thickness of 1400 nm and carrier concentration of 10^16^ cm^−3^, maximum PCE of 25.65% was recorded.

Table 3 shows a comparison of experimental and simulation results. Our results appear to be in satisfactory correlation with those previously reported. The distinctions between simulations and experiments are important to their methodology. There is a significant difference between experimental and simulation results because experiments are performed directly on the target machine, whereas software simulations are never performed in this manner. The experiments give confirmation of the object’s true behavior, with variable measurement errors, while simulated results provide insights based on similar theoretical models. As a result, the distinction is mostly between the real object and its theoretical and numerical descriptions, especially when other errors are greatly minimized.

## 4. Conclusions

Using the SCAPS-1D simulator, the performance of the proposed cell structure ITO/CeO_2_/SnS/Spiro-OMeTAD/Mo has been analyzed. To investigate the cell performance, distinct parameters of CeO_2_, Spiro-OMeTAD and SnS are optimized. The Spiro-OMeTAD made a considerable donation to the open-circuit voltage and efficiency. The higher carrier concentration of the CeO_2_ ETL was found to be beneficial to cell performance. The thickness of SnS appears to be a key component in determining cell performance. Due to their large impact on the resistance in series, CeO_2_/SnS density of interface defect and SnS defect density were optimized. The maximum recorded PCE with Spiro-OMeTAD HTL is 25.65% with J_SC_ of 33.74% mA/cm^2^, V_OC_ of 0.887 V and fill factor of 85.61%. Our result shows that SnS-based proposed device structure could prove to be an efficient device for low-cost and highly efficient thin-film solar cell in future work.

## Figures and Tables

**Table 1:** Physical parameters of ITO/CeO_2_/SnS/Spiro-OMeTAD/Mo heterostructure solar cell.

Parameters	ITO	CeO_2_	SnS	Spiro-OMeTAD
Thickness (nm)	50	100	1400	100
Band gap (eV)	3.6	3.5	1.31	3.2
Electron affinity (eV)	4.5	4.6	4.2	2.1
Dielectric permittivity (relative)	8.9	9	13	3
CB effective density of states (cm^−3^)	2.2 × 10^18^	1 × 10^20^	1.18 × 10^18^	2.5 × 10^18^
VB effective density of states (cm^−3^)	1.8 × 10^18^	2 × 10^21^	4.76 × 10^18^	1.8 × 10^19^
Electron mobility (cm^2^/Vs)	10	100	130	2 × 10^4^
Hole mobility (cm^2^/Vs)	10	25	4.3	2 × 10^4^
Shallow uniform donor density N_d_ (cm^−3^)	1 × 10^21^	1 × 10^21^	0	0
Shallow uniform acceptor density N_a_ (cm^−3^)	0	0	10^15^	1 × 10^20^
Electron thermal velocity (cm/s)	1 × 10^7^	1 × 10^7^	1 × 10^7^	1 × 10^7^
Hole thermal velocity (cm/s)	1 × 10^7^	1 × 10^7^	1 × 10^7^	1 × 10^7^
Defect density (cm^−3^)	0	1 × 10^14^	1 × 10^14^	
Radiative recombination coefficient (cm^3^/s)	0	2.3 × 10^−9^	2.3 × 10^−9^	2.3 × 10^−9^

**Table 2:** Interface values are employed in the device simulation.

Parameters	Spiro-OMeTAD/SnS Interface	CeO_2_/SnS Interface
Defect type	Neutral	Neutral
Capture cross-section electrons (cm^2^)	1 × 10^−19^	1 × 10^−19^
Capture cross-section holes (cm^2^)	1 × 10^−19^	1 × 10^−19^
Defect energy level E_t_	Above the highest E_v_	Above the highest E_v_
Energy with respect to a reference (eV)	0.06	0.06
Total density (cm^−2^)	1 × 10^10^	1 × 10^10^

**Table 3:** Comparison of physical parameters of various simulated and experimentally studied device structures.

Structures	V_OC_ V	J_SC_ mA/cm^2^	FF %	PCE %	References
SnS/Zn(O,S) (experimentally)	0.244	19.42	42.97	2.04	[19]
SnS/SnO_2_/Zn(O,S):N/ZnO (experimentally)	0.372	20.20	58.00	4.36	[4]
CZTS/SnS_2_/ZnO (simulated)	0.7178	26.99	65.67	12.73	[25]
Mo/SnS/CZTS/SnS_2_/ZnO (simulated)	0.9922	20.13	71.33	14.24	[26]
ZnO/CdS/CdTe/SnS/Ni (simulated)	0.845	26.46	84.50	21.83	[27]
p-SnS/CdS/n-Zn MgO (simulated)	~0.7	38.54	83	~23	[28]
ITO/CeO_2_/SnS/NiO/Mo (simulated)	0.890	32.67	86.19	25.06	[29]
ITO/CeO_2_/SnS/Spiro-OMeTAD (simulated)	0.887	33.74	85.61	25.65	This paper
